# Circulating Tumor DNA Mutations in Progressive Gastrointestinal Stromal Tumors Identify Biomarkers of Treatment Resistance and Uncover Potential Therapeutic Strategies

**DOI:** 10.3389/fonc.2022.840843

**Published:** 2022-02-22

**Authors:** Tun Kiat Ko, Elizabeth Lee, Cedric Chuan-Young Ng, Valerie Shiwen Yang, Mohamad Farid, Bin Tean Teh, Jason Yongsheng Chan, Nagavalli Somasundaram

**Affiliations:** ^1^ Laboratory of Cancer Epigenome, National Cancer Centre Singapore, Singapore, Singapore; ^2^ Cancer Discovery Hub, National Cancer Centre Singapore, Singapore, Singapore; ^3^ Division of Medical Oncology, National Cancer Centre Singapore, Singapore, Singapore; ^4^ Oncology Academic Clinical Program, Duke-NUS Medical School, Singapore, Singapore; ^5^ Institute of Molecular and Cell Biology, Singapore, Singapore; ^6^ Programme in Cancer and Stem Cell Biology, Duke-NUS Medical School, Singapore, Singapore

**Keywords:** liquid biopsy, imatinib, non-invasive, KIT, ctDNA (circulating tumor DNA)

## Abstract

Liquid biopsy circulating tumor DNA (ctDNA)-based approaches may represent a non-invasive means for molecular interrogation of gastrointestinal stromal tumors (GISTs). We deployed a customized 29-gene Archer^®^ LiquidPlex™ targeted panel on 64 plasma samples from 46 patients. The majority were known to harbor *KIT* mutations (*n* = 41, 89.1%), while 3 were *PDGFRA* exon 18 D842V mutants and the rest (*n* = 2) were wild type for *KIT* and *PDGFRA*. In terms of disease stage, 14 (30.4%) were localized GISTs that had undergone complete surgical resection while the rest (*n* = 32) were metastatic. Among ten patients, including 7 on tyrosine kinase inhibitors, with evidence of disease progression at study inclusion, mutations in ctDNA were detected in 7 cases (70%). Known somatic mutations in *KIT* (*n* = 5) or *PDGFRA* (*n* = 1) in ctDNA were identified only among 6 of the 10 patients. These *KIT* mutants included duplication, indels, and single-nucleotide variants. The median mutant AF in ctDNA was 11.0% (range, 0.38%–45.0%). In patients with metastatic progressive *KIT*-mutant GIST, tumor burden was higher with detectable *KIT* ctDNA mutation than in those without (median, 5.97 cm vs. 2.40 cm, *p* = 0.0195). None of the known tumor mutations were detected in ctDNA for localized cases (*n* = 14) or metastatic cases without evidence of disease progression (*n* = 22). In patients with serial samples along progression of disease, secondary acquired mutations, including a potentially actionable *PIK3CA* exon 9 c.1633G>A mutation, were detected. ctDNA mutations were not detectable when patients responded to a switch in TKI therapy. In conclusion, detection of GIST-related mutations in ctDNA using a customized targeted NGS panel represents an attractive non-invasive means to obtain clinically tractable information at the time of disease progression.

## Introduction

Gastrointestinal stromal tumor (GIST) is the commonest mesenchymal neoplasm originating from the gastrointestinal tract. GISTs are classically defined by activating oncogenic mutations in *KIT* (KIT proto-oncogene receptor tyrosine kinase) (80%) or *PDGFRA* (platelet-derived growth factor receptor alpha) (10%) genes (1–3). In advanced stages, most patients benefit from targeted therapy using the tyrosine kinase inhibitor (TKI) imatinib, though acquired resistance and disease progression associated with the development of secondary mutations usually occur after 18 to 24 months. Under pharmacological pressure, secondary mutations can develop, conferring resistance to imatinib. In imatinib-resistant GISTs, secondary mutations typically occur in the ATP-binding pocket (exon 13) or in the kinase activation loop (exon 17) (4).

In the contemporary management of patients with advanced GISTs, radiological response evaluation following TKI treatment remains standard of care, and there is currently no specific blood-based biomarker for the purposes of monitoring disease progression or to determine mechanisms responsible for acquired TKI resistance. At the time of disease progression, knowledge of the specific secondary mutations could enable tailoring of treatment, though this requires invasive tumor biopsy at the site of progression. Understandably, such an approach does not comprehensively capture the evolving global tumor landscape and may not yield viable results that reflect tumor heterogeneity (5). In addition to defining resistance pathways, the secondary mutations also have therapeutic implications. Previous preclinical studies have suggested that sunitinib is preferentially more active against mutations in the ATP-binding pocket, while regorafenib has increased activity against mutations in the kinase activation loop (6).

The optimal approach to determine mechanisms of acquired resistance during therapy and guide personalized approach to subsequent management remains to be elucidated. Non-invasive tumor mutation profiling using several liquid biopsy cell-free circulating tumor DNA (ctDNA)-based approaches has been explored in patients with GISTs (7). While some of these approaches have been shown to capture the molecular heterogeneity of the whole tumor, their utility is limited to patients with high tumor burden as a result of suboptimal assay sensitivity, and their practical use in the clinic remains in question. In this study, we investigated a liquid biopsy approach for the detection of primary and secondary acquired mutations in patients with GISTs, using a customized Archer^®^ LiquidPlex™ targeted panel. The advantage of Archer^®^ LiquidPlex™ includes the Anchored Multiplex PCR (AMP™) enrichment chemistry, in which ctDNA fragments are ligated to molecular barcodes that allow for error correction for confident variant reporting. Furthermore, Archer^®^ LiquidPlex™ is able to capture a fragment size that is smaller than 160 base pairs, a size that can be missed by other platforms. Finally, Archer^®^ LiquidPlex™ can detect variants at 0.3% allele frequency with ctDNA input that is as low as 1 ng.

## Patients and Methods

### Study Cohort

Blood samples were collected from patients who were diagnosed with GISTs and seen at the National Cancer Centre Singapore between April 1998 and September 2021. A total of 64 plasma samples and 7 whole blood samples from 46 patients were included in the final analysis. Relevant demographical and clinical information were collected and utilized for the analysis. For all GISTs diagnosed at our center, Sanger sequencing was routinely performed to detect *KIT* and *PDGFRA* gene mutations. Selected cases may undergo panel testing *via* next-generation sequencing at the discretion of the managing physician. All data were obtained at the time of diagnosis or subsequent follow-up. Written informed consent for use of biospecimens and clinical data was obtained in accordance with the Declaration of Helsinki. The research study was carried out with approval from the SingHealth Centralised Institutional Review Board (CIRB 2018/3182). The datasets created and analysed during this study are available from the corresponding authors upon reasonable request.

### Extraction and Quantification of ctDNA from Plasma and Whole Blood

Whole blood samples were collected in 10-ml EDTA-coated tubes (BD, Cat. 368589) and processed within 2 h of collection using an in-house protocol. The samples were centrifuged for 10 min at 300 × *g* at room temperature to separate plasma from red blood cells. Plasma layer was harvested in 1-ml aliquots in 1.5-ml Microcentrifuge Tubes (Axygen, MCT-150-C) and spun again in a microcentrifuge at 9,720 × *g* at 4°C. Plasma was collected and stored at −80°C until use. QIAamp Circulating Nucleic Acid Kit (Catalog #55114, Qiagen) was used to isolate ctDNA from plasma by following the manufacturer’s instruction. Purified ctDNA was stored at −20°C.

The following is the protocol for extracting ctDNA from frozen whole blood. DNA were isolated by using DNeasy Blood & Tissue Kit (Qiagen) with the following modification to the protocol. Briefly, proteinase K was added to whole blood at the following volume ratio (proteinase K:whole blood, 1:10). Subsequently, Buffer AL (with no ethanol added) was added into thawed blood, containing proteinase K, at a volume ratio of 6:1 before incubating at 56°C for at least 10 min. Absolute ethanol was added at a volume that was equal to that of Buffer AL used. The number of DNA purification columns used was dependent on the volume of whole blood. The ratio was 1 column per 200 µl of thawed whole blood. Subsequent steps followed the manufacturer’s instruction.

Once eluted, DNA was obtained from whole blood, Mag-Bind^®^ Total Pure NGS (Omega Bio-tek) paramagnetic beads were used to separate the genomic DNA from ctDNA; 0.6× bead volume (calculation was based on the volume of the DNA eluent) was added to the DNA eluent, and the beads were used to remove the genomic DNA. The beads containing the genomic DNA was isolated from the supernatant by a magnetic column. Subsequently, the leftover supernatant was moved to a clean microfuge tube where 2× bead volume (calculation was based on the volume of the original DNA eluent) was added. This volume of beads would isolate out the ctDNA from the supernatant. The beads were isolated by magnet, washed, and ctDNA was eluted according to the manufacturer’s instructions. The profile of the eluted ctDNA fraction was visualized on Agilent TapeStation by using Agilent genomic DNA ScreenTape (Agilent Technologies, CA, USA) to ensure that there was no contaminating genomic DNA. If genomic DNA was still present in the eluted ctDNA fraction, the DNA clean-up would be repeated. Purified ctDNA, from plasma or whole blood, were quantified by using Qubit dsDNA assay kit (Thermo Fisher Scientific). Purified ctDNA profile was also visualized on Agilent TapeStation by using Agilent High Sensitivity D100 ScreenTape.

### Next-Generation Sequencing Library Construction and Sequencing

NGS libraries were made from, depending on individual sample ctDNA yield, 5 ng to 37 ng (median: 10.1 ng) of ctDNA input. The NGS libraries were constructed by following the Archer^®^ LiquidPlex™ Protocol for Illumina^®^ (Invitae). We are using a customized Archer^®^ LiquidPlex™ targeted panel, dubbed LiquidPlex NCCS GIST 18265 v1.0, that consists of probes that target specific exons of 29 genes that are known to associate with cancer and encompass known somatic mutations of GISTs (**Supplementary Table 1**). The libraries were paired-end sequenced (2 × 150 bp) for 5–20 million raw reads with at least 5% PhiX by using the standard Illumina NGS protocol on the NovaSeq platform (Novogene).

### Bioinformatics Analysis

The raw sequencing data were analyzed with Archer Analysis pipeline (version 6.2.7; https://archerdx.com/technology-platform/analysis) as previously reported (8). We used the default settings for detecting variants that were statistically significant [in our case, the variant must have an allele frequency (AF) outlier *p*-value < 0.05]. For ctDNA samples where no significant variants were detected by the default setting, we would manually check all the mapped NGS reads for the presence of *KIT*, *PDGFRA*, or other variants that were previously detected by Sanger sequencing of the corresponding tumor. If such variant was detected, it would only be reported as a significant variant if AF outlier *p*-value < 0.05.

### Statistical Analysis

Comparisons of tumor size with detectable ctDNA were performed using Mann–Whitney *U* test. All statistical evaluations were made assuming a two-sided test with a significance level of 0.05 unless otherwise stated. All tests were performed using MedCalc statistical Software for Windows version 19.0.4 (MedCalc Software, Ostend, Belgium).

## Results

### Patient Characteristics

A total of 46 patients were included in the study (**Supplementary Figure 1**). The median age at study enrolment was 63.7 years (range, 30.3 to 89.1 years). Twenty-six (56.5%) were male and 20 (43.5%) were female. Primary tumor locations were stomach (*n* = 24, 52.2%), small bowel (*n* = 19, 41.3%), and rectum (*n* = 3, 6.5%). The majority were known to harbor *KIT* mutations (*n* = 41, 89.1%), while 3 patients (NCCS-GIST-06, NCCS-GIST-43, and NCCS-GIST-44) were *PDGFRA* exon 18 D842V mutants and *KIT* wild type. Two cases (NCCS-GIST-45 and NCCS-GIST-46) were wild type for both *KIT* and *PDGFRA*, one of which harbored *KRAS* exon2 G12V mutation. In terms of disease stage, 14 (30.4%) were localized GISTs that had undergone complete surgical resection. The rest (*n* = 32) were metastatic GISTs at various points of their treatment trajectories. Importantly, 10 patients, including 7 on TKI treatment, had evidence of disease progression at study inclusion and were the focus of the study. Clinical and demographic characteristics of all patients are summarized in **Table 1**.

**Table 1:** Characteristics of GIST patients at enrolment.

Characteristic	*N* (%)
*Total*	46 (100)
*Age at inclusion (years)*	
Median (range)	63.4 (30.3 to 89.1)
*Sex*	
Male	26 (56.5)
Female	20 (43.5)
*Primary tumor location*	
Stomach	24 (52.2)
Small intestine	19 (41.3)
Rectum	3 (6.5)
*Disease stage*	
Localized	14 (30.4)
Metastatic	32 (69.6)
*Evidence of progression*	
No	36 (78.3)
Yes	10 (21.7)
*Known somatic mutations*	
KIT†	41 (89.1)
PDGFRA (exon 18 D842V)	3 (6.5)
KIT/PDGFR wild-type‡	2 (4.3)
Adjuvant therapy (localized)*	
Imatinib	5 (35.7)
None	9 (64.3)
Palliative therapy (metastatic)*	
Imatinib	18 (56.3)
Sunitinib	2 (6.3)
Pazopanib	1 (3.1)
Avapritinib	1 (3.1)
None	10 (31.3)

†Exon 11 (n = 29), exons 11 and 13 (n = 1), exons 11 and 17 (n = 2), exon 9 (n = 5), site unknown (n = 4).

‡One case of KIT/PDGFRA wild type known to harbor KRAS Exon2, c.35G>T/p.Gly12Val.

*Treatment at time of study inclusion.

### Molecular Profiling Using ctDNA

**Figure 1:**
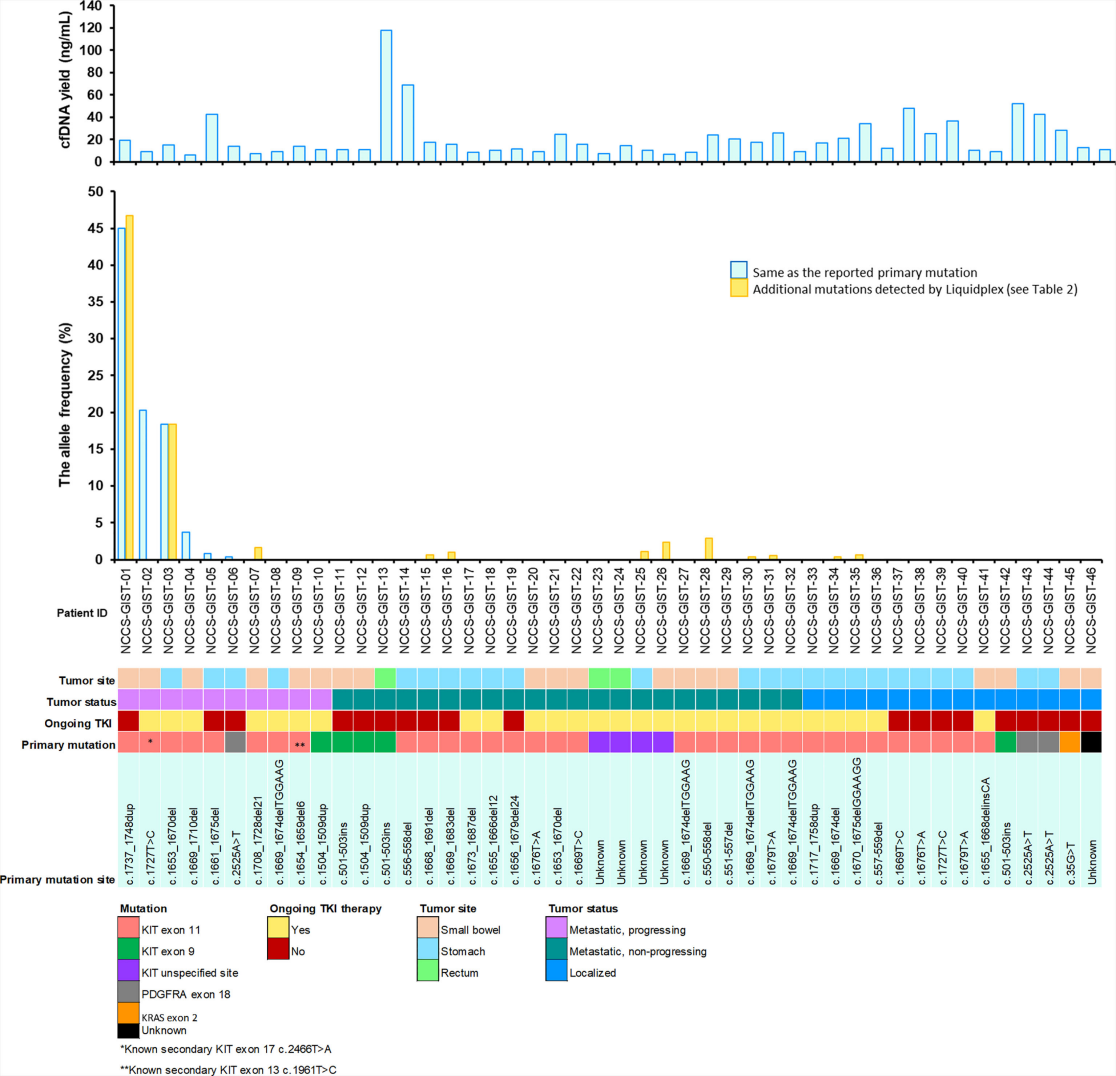
Overview of study cohort and plasma ctDNA mutation detection. Clinical and molecular characteristics of the 46 patients included in the study are shown. For the mutant allele frequency bar chart, the light blue bar 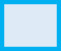 indicates mutants that were detected in ctDNA are the same as that reported for the corresponding primary tumor. The gold-colored bar 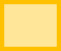 indicates additional mutants detected in ctDNA that is not reported in the corresponding primary tumor. See **Table 2** for more information on the mutation profiles of both the primary tumor and the corresponding ctDNA.

**Figure 2:**
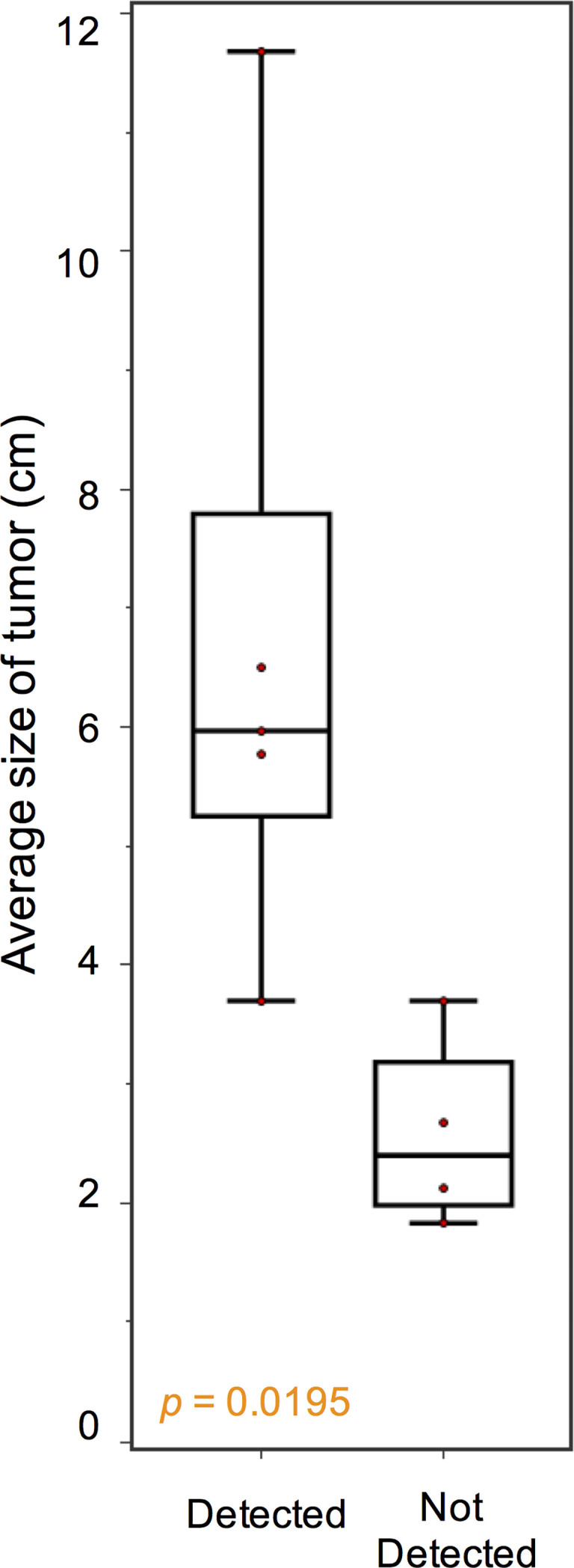
Association between *KIT* ctDNA mutation detection and tumor burden. In patients with advanced progressive GIST, tumor burden, as measured by the average diameter of the 3 largest lesions, was higher with detectable *KIT* ctDNA mutation than in those without (median, 5.97 cm vs. 2.40 cm, *p* = 0.0195).

Blood samples of the 46 patients were drawn at study inclusion. Targeted exon panel sequencing was performed to identify mutations in plasma ctDNA. Among the 10 patients with metastatic GIST with evidence of disease progression, mutations in ctDNA were detected in 7 cases (70%). Known somatic mutations in *KIT* (*n* = 5) or *PDGFRA* (*n* = 1) in ctDNA were identified only among 6 of the 10 patients (**Figure 1**). These *KIT* mutants included short sequence tandem duplication, indels, and single-nucleotide variants. The median mutant AF in ctDNA was 11.0% (range 0.38%–45.0%). In patients with metastatic progressive *KIT*-mutant GIST, tumor burden (as measured by the average diameter of the 3 largest lesions) was higher with detectable *KIT* ctDNA mutation than in those without (median, 5.97 cm vs. 2.40 cm, *p* = 0.0195) (**Figure 2**). The 4 cases with undetectable primary *KIT* mutations include GISTs with exon 11 c.1708_1728del (NCCS-GIST-07), exon 11 c.1669_1674del (NCCS-GIST-08), exon 11 c.1654_1659del and exon 13 c.1961T>C (NCCS-GIST-09), and exon 9 c.1504_1509dup (NCCS-GIST-10). None of the known tumor mutations were detected in ctDNA for localized cases (*n* = 14) or metastatic cases without evidence of disease progression (*n* = 22). In patient NCCS-GIST-02, only the known *KIT* exon 11 c.1727T>C mutation, but not the exon 17 c.2466T>A mutation was detected in ctDNA. On the other hand, in patient NCCS-GIST-03, ctDNA identified *KIT* exon 17 c.2467T>G on top of the known exon 11 c.1653_1670del mutation—this patient had prior imatinib-resistant GIST (no molecular evaluation done apart from time of diagnosis) and was progressing despite the 4th-line treatment with pazopanib. For patient NCCS-GIST-07 with small bowel GIST progressing on 1st-line treatment with imatinib, ctDNA detected *TP53* c.879_880del and *SETD2* c.2238del mutations, but not the known *KIT* exon 11 c.1708_1728del mutation (**Table 2**).

**Table 2:** Mutational profiles of tumor and plasma ctDNA.

Patient ID	Primary site	Disease status	Treatment at time of study inclusion	Known tumor mutation	Mutation in ctDNA	Allele frequency
NCCS-GIST-01	Small bowel	Metastatic^†^	None	KIT Exon 11 c.1737_1748dup	KIT Exon 11 c.1737_1748dup	45
					SETD2 c.1844C>T	46.7
NCCS-GIST-02	Small bowel	Metastatic^†^	Avapritinib	KIT Exon 11 c.1727T>C	KIT Exon 11 c.1727T>C	20.3
				KIT Exon 17 c.2466T>A		
NCCS-GIST-03	Gastric	Metastatic^†^	Pazopanib	KIT Exon 11 c.1653_1670del	KIT Exon 11 c.1653_1670del	18.4
					KIT Exon 17 c.2467T>G	20.3
NCCS-GIST-04	Small bowel	Metastatic^†^	Imatinib	KIT Exon 11 c.1669_1710del	KIT Exon 11 c.1669_1710del	3.68
NCCS-GIST-05	Gastric	Metastatic^†^	None	Kit Exon 11 c.1661_1675del	Kit Exon 11 c.1661_1675del	0.81
NCCS-GIST-06	Gastric	Metastatic^†^	None	PDGFRA Exon 18 c.2525A>T	PDGFRA Exon 18 c.2525A>T	0.38
NCCS-GIST-07	Small bowel	Metastatic^†^	Imatinib	KIT Exon 11 c.1708_1728del	TP53 Exon 8 c.879_880del	1.68
					SETD2 c.2238del	1.32
NCCS-GIST-08	Gastric	Metastatic^†^	Imatinib	KIT Exon 11 c.1669_1674del		
NCCS-GIST-09	Small bowel	Metastatic^†^	Sunitinib	KIT Exon 11 c.1654_1659del		
				KIT Exon 13 c.1961T>C		
NCCS-GIST-10	Small bowel	Metastatic^†^	Imatinib	KIT Exon 9 c.1504_1509dup		
NCCS-GIST-11	Small bowel	Metastatic	None	KIT Exon 9 c.501-503ins		
NCCS-GIST-12	Small bowel	Metastatic	None	KIT Exon 9 c.1504_1509dup		
NCCS-GIST-13	Rectum	Metastatic	None	KIT Exon 9 c.501-503ins		
NCCS-GIST-14	Gastric	Metastatic	None	KIT Exon 11 c.556-558del		
NCCS-GIST-15	Gastric	Metastatic	None	KIT Exon 11 c.1668_1691del	IDH2 c.435dup	0.68
NCCS-GIST-16	Gastric	Metastatic	None	KIT Exon 11 c.1669_1683del	IDH2 c.435dup	1.03
NCCS-GIST-17	Gastric	Metastatic	Imatinib	KIT Exon 11 c.1673_1687del		
NCCS-GIST-18	Gastric	Metastatic	Imatinib	KIT Exon 11 c.1655_1666del		
NCCS-GIST-19	Gastric	Metastatic	None	KIT Exon 11 c.1656_1679del		
NCCS-GIST-20	Small bowel	Metastatic	Imatinib	KIT Exon 11 c.1676T>A		
NCCS-GIST-21	Small bowel	Metastatic	Imatinib	KIT Exon 11 c.1653_1670del		
NCCS-GIST-22	Small bowel	Metastatic	Imatinib	KIT Exon 11 c.1669T>C		
				TP53 Exon 8 c.841G>A		
NCCS-GIST-23	Rectum	Metastatic	Imatinib	KIT (site unknown)		
NCCS-GIST-24	Rectum	Metastatic	Imatinib	KIT (site unknown)		
NCCS-GIST-25	Gastric	Metastatic	Imatinib	KIT (site unknown)	KRAS c.194G>A	1.1
NCCS-GIST-26	Small bowel	Metastatic	Imatinib	KIT (site unknown)	KRAS c.175G>A	1.62
					KRAS c.186_187delins	2.33
NCCS-GIST-27	Small bowel	Metastatic	Imatinib	KIT Exon 11 c.1669_1674del		
NCCS-GIST-28	Small bowel	Metastatic	Imatinib	KIT Exon 11 c.550-558del	KIT Exon 11 c.1650_1667del	0.65
					KIT Exon 11 c.1667A>T	0.57
					KRAS c.175G>A	1.16
					KRAS c.194G>A	2.9
					KRAS c.186_187delins	1.7
NCCS-GIST-29	Small bowel	Metastatic	Sunitinib	KIT Exon 11 c.551-557del		
NCCS-GIST-30	Gastric	Metastatic	Imatinib	KIT Exon 11 c.1669_1674del	KIT Exon 11 c.1706T>G	0.36
NCCS-GIST-31	Gastric	Metastatic	Imatinib	KIT Exon 11 c.1679T>A	SETD2 c.3948del	0.52
NCCS-GIST-32	Gastric	Metastatic	Imatinib	KIT Exon 11 c.1669_1674del		
NCCS-GIST-33	Gastric	Localized	Imatinib	KIT Exon 11 c.1717_1758dup		
NCCS-GIST-34	Gastric	Localized	Imatinib	KIT Exon 11 c.1669_1674del	KIT Exon 11 c.1706T>G	0.36
NCCS-GIST-35	Gastric	Localized	Imatinib	KIT Exon 11 c.1670_1675del	IDH2 c.435dup	0.67
NCCS-GIST-36	Gastric	Localized	Imatinib	KIT Exon 11 c.557-559del		
NCCS-GIST-37	Gastric	Localized	None	KIT Exon 11 c.1669T>C		
NCCS-GIST-38	Gastric	Localized	None	KIT Exon 11 c.1676T>A		
NCCS-GIST-39	Gastric	Localized	None	KIT Exon 11 c.1727T>C		
NCCS-GIST-40	Gastric	Localized	None	KIT Exon 11 c.1679T>A		
NCCS-GIST-41	Small bowel	Localized	Imatinib	KIT Exon 11 c.1655_1668delins		
NCCS-GIST-42	Small bowel	Localized	None	KIT Exon 9 c.501-503ins		
NCCS-GIST-43	Gastric	Localized	None	PDGFRA Exon 18 c.2525A>T		
NCCS-GIST-44	Gastric	Localized	None	PDGFRA Exon 18 c.2525A>T		
NCCS-GIST-45	Small bowel	Localized	None	KRAS Exon2 c.35G>T		
NCCS-GIST-46	Small bowel	Localized	None	KIT/PDGFRA wild-type		

†Evidence of progression.

Mutations in *SETD2*, previously reported to confer worse prognosis in GIST (9), were detected in ctDNA in 3 patients with metastatic GIST. In patients with localized GIST following surgical resection (*n* = 14), including 5 on adjuvant imatinib, no known tumor mutations could be detected from ctDNA. Likewise, none could be detected from ctDNA from patients with metastatic GIST without evidence of disease progression (*n* = 22), including 15 patients on TKI treatment. In this group of patients, mutations in *IDH2* (*n* = 3), *KRAS* (*n* =3), *KIT* (*n* = 3), and *SETD2* (*n* = 1) were identified at low AF (median, 0.86%; range, 0.68 to 2.9%), though the corresponding mutation in the primary tumor was not known.

### ctDNA Mutation Detection in Patients Progressing on Treatment

**Figure 3:**
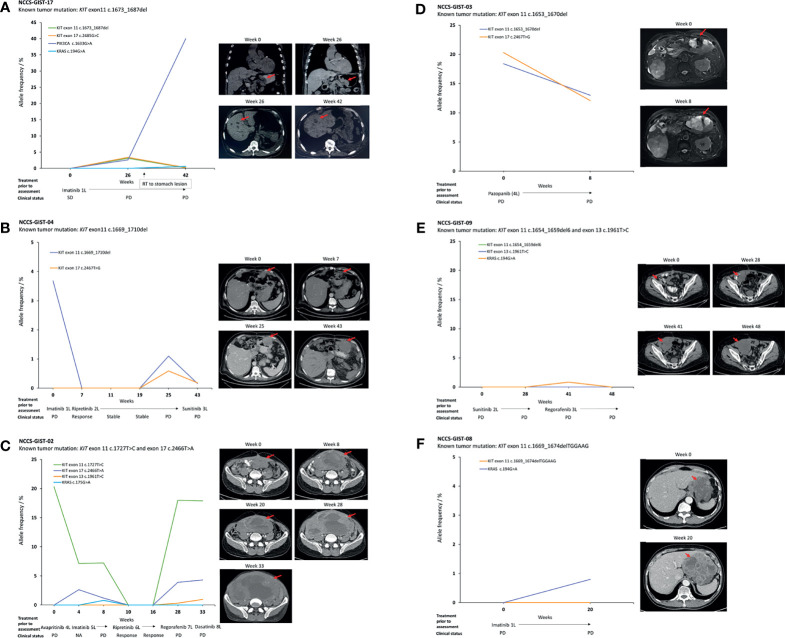
Temporal correlation of ctDNA mutations and patients with advanced GIST progressing while on TKI therapy. **(A)** Detectable *KIT* exon 11 and 17 mutations, as well as a potentially actionable *PI3K3CA* c.1633G>A mutation upon disease progression. The *PI3K3CA* variant was dominant over other mutations in ctDNA on further disease progression. **(B–D)** Trajectory and correlation of ctDNA variants with disease status. **(E, F)** Non-detection of *KIT* mutations in ctDNA despite disease progression. Each figure is accompanied with the CT scans taken at the indicated time points. The red arrows refer to the location of the tumor. SD, stable disease; PD progressive disease.

Serial plasma samples were available for 6 patients with metastatic GIST who underwent TKI therapy. For NCCS-GIST-17 (**Figure 3A**), the *KIT* exon 11 c.1673_1687del variant, as detected in the tumor, was not detected in the ctDNA from the first plasma sampling when no disease progression was evident (stable disease, SD) (week 0). In the ctDNA from the second plasma sampling at the time of disease progression in the primary stomach tumor (week 26), 2 *KIT* variants were detected, namely, exon 11 c.1673_1687del (AF = 3.1%) and exon 17 c.2485G>C (AF = 3.4%), the latter of which was a new variant not previously detected in the tumor. Additionally, a *PIK3CA* c.1633G>A variant was also detected (AF = 2.6%). Interestingly, in the ctDNA from the third plasma sampling (week 42, with progressive liver metastases), the *PIK3CA* c.1633G>A variant AF increased by nearly 32-fold (AF = 82.5%) while those *KIT* variants were reduced by at least 14.2-fold (exon 11 c.1673_1687del, AF = 0.15%; exon 17 c.2485G>C, AF = 0.24%). Generally, the detection of the *KIT* and *PIK3CA* variants in the ctDNA positively correlated with disease progression as evaluated by computed tomography (CT) scans.

For patients NCCS-GIST-04, NCCS-GIST-02, and NCCS-GIST-09, multiple plasma samplings at different time points (4–7 samples) have allowed for the monitoring of dynamic changes to the different *KIT* variants detected in the ctDNA in response to different types of TKI used. In NCCS-GIST-04 (**Figure 3B**) and NCCS-GIST-02 (**Figure 3C**), *KIT* variants detected in ctDNA progressively decreased and were not detected in ctDNA when patients were responsive to TKI treatments. However, we observed that after a period of positive drug response, known *KIT* variants re-emerged in ctDNA with additional new variants that were not previously detected in tumor. The emergence of additional new *KIT* variant correlated with resistance to corresponding TKI treatment and disease progression.

Patient NCCS-GIST-04 switched from imatinib to ripretinib (at week 0) at the time of disease progression based on imaging assessment (**Figure 3B**). Imaging at week 7 confirmed response to ripretinib therapy—correspondingly, the AF for the *KIT* exon 11 c.1669_1710del variant fell from 3.68% at week 0 to 0% at week 7. Subsequently from week 7 to week 19, no *KIT* variant was detected in the ctDNA, and this coincided with a state of continued disease stability for the patient. At week 25, disease progression was detected on CT scan, and this coincided with the re-emergence of the *KIT* variant exon 11 c.1669_1710del (AF = 1.1%) and the detection of a new *KIT* variant exon 17 c.2467T>G (AF = 0.59%). At week 43, the AF values for both *KIT* variants were still detectable, albeit reduced (exon 11 c.1669_1710del, AF = 0.16%; exon 17 c.2467T>G, AF = 0.18%). This coincided with the patient switching to sunitinib. Despite the reduction in AF values for the 2 *KIT* variants, the CT scan showed that the disease had progressed with increased tumor size.

On the other hand, NCCS-GIST-02 switched from avapritinib to imatinib and subsequently ripretinib as a result of lack of response in terms of tumor reduction in the first 8 weeks of monitoring; the *KIT* variants from exon 11 c.1727T>C and exon 17 c.2466T>A became undetectable from week 10 to week 16 (**Figure 3C**), correlating with positive response to ripretinib treatment. Subsequently, ctDNA collected from week 28 and week 33 contain not only the previously detected *KIT* variants from exon 11 c.1727T>C and exon 17 c.2466T>A, but also a new exon 13 c.1961T>C variant. This indicated the emergence of a novel TKI-resistant variant that correlated with disease progression. Conversely, for NCCS-GIST-09 (**Figure 3E**), despite disease progression, no *KIT* variant, as previously detected in tumor, was detected in ctDNA collected at the different time points. There was, however, a *KRAS* c.194G>A variant that was detected at week 41 albeit at low AF (0.84%), but it was not detected at the last sampling at week 48.

There were 2 patients with 2 serial samplings, namely, NCCS-GIST-03 and NCCS-GIST-08. For NCCS-GIST-03, 2 *KIT* variants were detected (**Figure 3D**). The exon 11 c.1653_1670del variant was previously detected in tumor, while the exon 17 c.2467T>G variant was only found in plasma ctDNA. Although the variant AF for both variants decreased from week 0 to week 8, it did not correlate with decrease in tumor size. For NCCS-GIST-08 (**Figure 3F**), the *KIT* variant previously reported in tumor was not detected in ctDNA from both samplings (week 0 and week 20). A *KRAS* c.194G>A variant was detected, albeit at low AF (0.8%), in the week 20 sampling.

### ctDNA Mutation Detection in Whole Blood Samples

In an exploratory analysis, we attempted to detect known variants (previously found in the tumor) from ctDNA extracted from whole blood rather than from plasma. ctDNA was extracted from whole blood from 7 patients with metastatic GIST (**Supplementary Table 2**). NGS libraries were subsequently constructed from these extracted ctDNA. We compared the result from whole blood with those corresponding plasma-derived ctDNA as well as tumor. *KIT* variant was detected in 1 whole blood sample from NCCS-GIST-02. The *KIT* variant, exon 11 c.1727T>C, was the same as that reported for the corresponding plasma-derived ctDNA and tumor. The AF for this *KIT* variant in whole blood-derived ctDNA was 1.61%. This AF value was approximately 1/12 of the AF value found in plasma ctDNA (20.3%).

## Discussion

Our results showed that known tumor mutations, including both *KIT* and *PDGFRA*, were detectable in ctDNA only among patients with metastatic GIST with measurable disease progression. However, even in this group of patients, the detection of these mutations may depend on tumor burden at the time of progression. In some cases (NCCS-GIST-08 and NCCS-GIST-09), *KIT* mutations in ctDNA remain undetectable despite continued disease progression across serial samplings, suggesting that not all GISTs shed sufficient ctDNA for detection of mutations. Interestingly, as demonstrated by case NCCS-GIST-02 at week 0, despite the primary *KIT* exon 11 c.1727T>C being detected at high AF, the secondary *KIT* exon 17 c.2466T>A was not detected, implying that these mutations may not be shed at the same rates. Subsequent sampling upon disease progression simultaneously identified *KIT* exon 17 c.2466T>A and exon 13 c.1961T>C variants. Regardless, any detected ctDNA mutation became undetectable upon disease response to a subsequent line of TKI therapy. These results are generally consistent with previous reports (10–13). Taken together, these findings highlight the value of ctDNA mutation testing in the setting of progressive disease, enabling the detection of secondary acquired mutations, and facilitating treatment response assessment.

Among the GIST patients who progressed on TKI therapy evaluated in our study, we identified a potentially actionable acquired *PIK3CA* exon 9 c.1633G>A variant in NCCS-GIST-17 upon resistance to imatinib. In a previous study on 529 imatinib-naïve GISTs, only eight primary and two metastatic cases harbored *PIK3CA* mutations, though these cases tended to be large (>10 cm) (14). These results suggest that *PIK3CA* mutations may confer growth advantages in GISTs and may form the dominant clone in the setting of imatinib resistance. Consequently, this offers an opportunity for therapeutic intervention using PI3K inhibitors (15). Interestingly, we also observed the occurrence of mutations in the histone modifier gene *SETD2* in three patients. Previously, Huang et al. demonstrated somatic alterations of *SETD2* in 10 out of 89 (11.2%) high-risk/metastatic GIST cases but not low-/intermediate-risk cases. In gastric GISTs, *SETD2* mutations were associated with hypomethylated heterochromatin and worse relapse-free survival (9). The ability to identify predictive and prognostic biomarkers in a non-invasive manner is an attractive feature of liquid biopsy-based testing, though the actual clinical utility will require validation in a larger cohort.

Several liquid biopsy-based assays deployed for detection of *KIT* or *PDGFRA* mutations have previously been reported in GISTs, including allele-specific ligation PCR (10), digital droplet PCR (11), BEAMing (16), and targeted amplicon sequencing (12, 17). Although assays targeting single mutations are highly sensitive, they are not easily generalizable in GISTs as they are characterized by a range of primary and secondary mutations. In our study, we deployed a customized targeted panel (Archer^®^ LiquidPlex™) comprising 29 cancer and/or GIST-associated genes, emphasizing the utility of a larger sequencing footprint in picking up clinically relevant mutations beyond *KIT* and *PDGFRA*. Similar to a previous report (18), the sensitivity of our assay depends on tumor size, and is most applicable in the setting of progressive disease and TKI resistance. An additional limiting factor in the detection of ctDNA in our study may include the extent of vascularization of individual tumors, which would determine the ease of accessibility into the blood system for the ctDNA. We do take note that some of our plasma samples have been in storage at −80°C for more than the latest recommended storage period of 9 months (19). Among the 7 out of 10 patients with progressing metastatic GIST with significant detectable ctDNA mutations, 4 of the plasma samples were stored between 8 and 10 years, while the rest (*n* = 3) were stored for less than 1 year. In this case, storage period may not be the main issue. It is likely that when stored properly at the right temperature and repeated freeze–thaw is not allowed, plasma can be stored for a longer period. A previous report suggests the feasibility of using small volumes of dried whole blood spots for ctDNA mutation detection (20), and our exploratory investigation showed that this approach is clearly less sensitive than using plasma ctDNA, and the reagent costs also implies lower cost-effectiveness.

In conclusion, detection of GIST-related mutations in ctDNA using a customized targeted NGS panel represents an attractive non-invasive means to obtain clinically tractable information.

## Data Availability Statement

The original contributions presented in the study are included in the article/**Supplementary Material**. Further inquiries can be directed to the corresponding authors.

## Ethics Statement

The studies involving human participants were reviewed and approved by SingHealth Centralised Institutional Review Board. The patients/participants provided their written informed consent to participate in this study.

## Author Contributions

TK and JC analyzed the data and drafted the manuscript. TK, EL, CN, and BT provided technical advice and experimental support. VY, MF, and JC obtained patient data and contributed samples. JC and NS designed the study, interpreted the results, and revised the manuscript. All authors contributed to the article and approved the submitted version.

## Funding

This work was supported by the ArcherDX Merit Grant Award (ArcherDX LLC is a subsidiary of Invitae Corporation), Singapore Ministry of Health’s National Medical Research Council Research Training Fellowship (NMRC/Fellowship/0054/2017), SHF-Foundation Research Grant (SHF/FG653P/2017), Agency for Science, Technology and Research (A*STAR) IAF-PP Grant (H18/01/a0/019), as well as the SingHealth Duke-NUS Academic Medical Centre and Oncology ACP (08-FY2017/P1/14-A28, 08/FY2020/EX/08-A43, and 08/FY2020/EX/75-A151). The funder was not involved in the study design, collection, analysis, interpretation of data, the writing of this article or the decision to submit it for publication.

## Conflict of Interest

The authors declare that the research was conducted in the absence of any commercial or financial relationships that could be construed as a potential conflict of interest.

## Publisher’s Note

All claims expressed in this article are solely those of the authors and do not necessarily represent those of their affiliated organizations, or those of the publisher, the editors and the reviewers. Any product that may be evaluated in this article, or claim that may be made by its manufacturer, is not guaranteed or endorsed by the publisher.
