# Supplementary material for: Circulating Tumor DNA Mutations in Progressive Gastrointestinal Stromal Tumors Identify Biomarkers of Treatment Resistance and Uncover Potential Therapeutic Strategies
Source: Front Oncol. 2022 Feb 22;12:840843. doi: 10.3389/fonc.2022.840843 (PMC8904145; doi:10.3389/fonc.2022.840843)
Supplement: Supplementary file 1 [file DataSheet_1.pdf]

Supplementary Figure 1. A flow chart that show the number of patient in this study as well as the primary tumor mutation status for *KIT*, *PDGFRA* & *KRAS* genes. Additionally, the disease status (localized or metastatic) for these patients are also indicated.

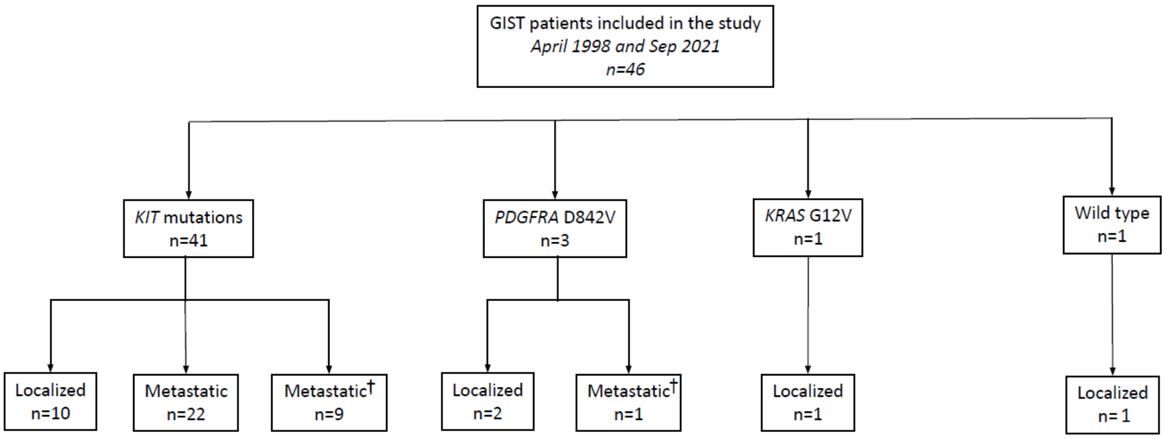

†Evidence of progression
