# Supplementary material for: Circulating Tumor DNA Mutations in Progressive Gastrointestinal Stromal Tumors Identify Biomarkers of Treatment Resistance and Uncover Potential Therapeutic Strategies
Source: Front Oncol. 2022 Feb 22;12:840843. doi: 10.3389/fonc.2022.840843 (PMC8904145; doi:10.3389/fonc.2022.840843)
Supplement: Supplementary file 2 [file Table_1.docx]

**Supplementary Table 1. Customized Archer® LiquidPlex™ targeted panel**

| **LiquidPlex NCCS GIST 18265 v1.0** | | |
| --- | --- | --- |
| **Gene** | **Accession** | **Target Exon** |
| AKT1 | NM_005163 | 3 |
| ALK | NM_004304 | 22,23,25 |
| AR | NM_033031 | 4,5,8 |
| BRAF | NM_004333 | 11,15 |
| CTNNB1 | NM_001904 | 3 |
| DDR2 | NM_006182 | 17 |
| EGFR | NM_005228 | 12,18,19,20,21 |
| ERBB2 | NM_004448 | 8,20 |
| ESR1 | NM_000125 | 5,7,8 |
| FGFR1 | NM_015850 | 13 |
| HRAS | NM_005343 | 2,3 |
| IDH1 | NM_005896 | 4 |
| IDH2 | NM_002168 | 4 |
| KIT | NM_000222 | 9,11,13,17,18 |
| KRAS | NM_004985 | 2,3,4 |
| MAP2K1 | NM_002755 | 2,3 |
| MAP2K2 | NM_030662 | 3 |
| MET | NM_000245 | 14 |
| MTOR | NM_004958 | 44,45,50 |
| NRAS | NM_002524 | 2,3 |
| NTRK1 | NM_002529 | 14,15 |
| NTRK3 | NM_002530 | 16,17 |
| PDGFRA | NM_006206 | 12,14,16,18 |
| PIK3CA | NM_006218 | 10,21 |
| RET | NM_020630 | 11,13,14,15,16 |
| ROS1 | NM_002944 | 38,40 |
| SETD2 | NM_014159 | All exons |
| SMAD4 | NM_005359 | 9 |
| TP53 | NM_000546 | All exons |

**Supplementary Table 2. Detection of GIST mutations in whole blood**

| **Patient ID** | **Mutation in ctDNA** | **Allele fraction (%)** | **ctDNA in whole blood (ng/ml)** | **Comment** |
| --- | --- | --- | --- | --- |
| NCCS-GIST-02 | KIT Exon 11 c.1727T>C | 1.61 | 32.5 | Variant *KIT* Exon 11 c.1727T>C detected was the same as tumor and plasma ctDNA. AF for *KIT* variant for whole blood is about 8% of that from plasma ctDNA |
| NCCS-GIST-04 | No significant variant detected |  | 4.54 |  |
| NCCS-GIST-08 | No significant variant detected |  | 7.4 |  |
| NCCS-GIST-09 | No significant variant detected |  | 0.91 |  |
| NCCS-GIST-10 | No significant variant detected |  | 31.7 |  |
| NCCS-GIST-19 | No significant variant detected |  | 91 |  |
| NCCS-GIST-21 | No significant variant detected |  | 1.25 |  |
